# Solvent-Dependent Green Synthesis of ZnO Nanopowders Using *Mitragyna speciosa* Leaf Extract: Impact on Piezo-Photocatalytic and Antibacterial

**DOI:** 10.3390/gels12070596

**Published:** 2026-07-03

**Authors:** Thanyapa Sanyen, Maneerat Songpanit, Thanaphon Kansaard, Supamas Wirunchit, Sutee Chutipaijit, Keiichi N. Ishihara, Hideyuki Okumura, Wisanu Pecharapa, Wanichaya Mekprasart, Kanokthip Boonyarattanakalin

**Affiliations:** 1 Department of Nanoscience and Nanotechnology, School of Integrated Innovative Technology, King Mongkut’s Institute of Technology Ladkrabang, Ladkrabang, Bangkok 10520, Thailand; 67116003@kmitl.ac.th (T.S.); 66116007@kmitl.ac.th (M.S.); thanaphon.ka@kmitl.ac.th (T.K.); supamas.wi@kmitl.ac.th (S.W.); sutee.ch@kmitl.ac.th (S.C.); wisanu.pe@kmitl.ac.th (W.P.); kanokthip.bo@kmitl.ac.th (K.B.); 2 Open Innovation Institute, Kyoto University, Yoshida Honmachi, Sakyo, Kyoto 606-8501, Japan; ishihara.keiichi.6w@kyoto-u.ac.jp; 3 Graduate School of Energy Science, Kyoto University, Yoshida Honmachi, Sakyo, Kyoto 606-8501, Japan; okumura.hideyuki.4e@kyoto-u.ac.jp

**Keywords:** antibacterial, green synthesis, *Mitragyna speciosa*, piezo-photocatalytic, zinc oxide

## Abstract

ZnO nanopowders were synthesized via a solvent-mediated green route using *Mitragyna speciosa* Korth. leaf extract as reducing and stabilizing agents. Deionized water and methanol were employed to tailor the phytochemical composition of the extracts. The influence of extract concentration (5–20 mL) and solvent polarity on structural, morphological, and functional properties was systematically investigated. Structural analyses confirmed the formation of Zn-O bonds and a phase-pure hexagonal wurtzite ZnO without secondary phases. Surface morphology revealed solvent-dependent morphological evolution toward spherical shapes and reduced aggregation in the methanol-derived system. For antibacterial activity, green ZnO nanopowders demonstrated enhanced biocidal effects against Gram-negative *Escherichia coli* and Gram-positive *Staphylococcus aureus*, comparable to that of commercial ZnO nanoparticles. Enhanced piezo-photocatalytic degradation of rhodamine B was achieved under combined light and ultrasonic irradiation, with superior performance observed for methanol-derived ZnO. This enhancement is attributed to the synergistic interplay of solvent-induced defect states, reduced particle size, and piezoelectric field-driven charge separation. Scavenger analysis confirmed that superoxide radicals (${\cdot O}_{2}^{-}$) dominate the degradation pathway by green-synthesized ZnO nanopowders prepared from different solvent extracts. Thus, a correlation between solvent-mediated phytochemical environments and piezo-photocatalytic activity provides new insights for the design of sustainable, high-performance ZnO-based catalysts.

## 1. Introduction

Nowadays, nano-scaled materials have garnered significant attention due to their novel structural properties and unique functionalities. Various physical and chemical techniques have been used in nanomaterial synthesis, enabling the production of diverse nanostructures. However, these conventional methods often involve environmentally hazardous procedures, high costs, and heat treatment processes. As an alternative, green synthesis methods offer an environmentally friendly, simple procedure, and biocompatible approach to nanomaterial production. This method is particularly suitable for applications in specific fields, such as pharmaceuticals and biotechnology [1]. The in vitro green synthesis of nanoparticles is an effective approach for developing materials with desired properties. A key aspect of this technique is the utilization of internal biomolecules derived from plants and microorganisms, which act as reducing and chelating agents. These biomolecules derived from natural extracts have attracted considerable attention in green nanotechnology due to their environmental compatibility, low toxicity, renewability, and cost-effectiveness. Unlike conventional chemical reducing agents, which may generate hazardous by-products, naturally derived phytochemicals provide a sustainable alternative for nanoparticle synthesis. The bioactive compounds contain functional groups capable of interacting with metal ions, facilitating nanoparticle formation and stabilization. In addition to their reducing capability, phytochemicals play an important role as natural chelating agents [2]. Various plant extracts, rich in polyphenols, flavonoids, stilbenes, and other bioactive compounds, have been widely used as natural reducing and capping agents for the green synthesis of nanomaterials. These natural components in the extraction process promote the formation of nanomaterials, thereby minimizing the use of harmful chemicals during synthesis. In addition, plant extracts are increasingly recognized as naturally occurring soft-matter systems because their biomolecular constituents, including polysaccharides, proteins, and polyphenols, can establish hydrogen-bonded molecular networks and other non-covalent interactions, exhibiting gel-like characteristics [3]. Consequently, natural polymers and phytochemical-rich extracts have attracted increasing attention in gel-based materials owing to their biocompatibility, biodegradability, and abundant functional groups suitable for biomedical, antioxidant, and antimicrobial applications. For example, aloe vera leaf extract was chosen as a reducing agent via biosynthesized sol–gel method to produce amorphous Fe_2_O_3_ nanoflakes [4]. An aqueous extract of *Durio zibethinus* L. (Durian) seed, containing saccharides and amino acids, has been employed for the green synthesis of silver nanoparticles. These biomolecules act as reducing and natural capping agents for Ag^+^ ions, resulting in stable silver nanoparticles with excellent antimicrobial and photocatalytic activity [5]. The aqueous extract of sea lavender, *Limonium pruinosum* (L.) Chaz., was used as a reducing, capping, and stabilizing agent to obtain zinc oxide nanoparticles with potent antibacterial and antifungal activity [6]. Meanwhile, *Mitragyna speciosa* Korth., commonly known as Kratom in Thailand, belongs to the *Rubiaceae* family and is widely distributed across Southeast Asia, including Thailand, Malaysia, and Indonesia. This plant has long been utilized in traditional medicine, particularly for its analgesic and anti-diarrheal properties [7]. The leaves of *Mitragyna speciosa* are rich in bioactive compounds, for example, flavonoids, alkaloids, terpenoid saponins, and polyphenols, which can act as reducing and stabilizing agents in material synthesis [8]. In addition to their reducing capability, phytochemicals play an important role as natural chelating agents. Functional groups such as hydroxyl (–OH), carbonyl (C=O), and amine (–NH_2_) can coordinate with metal ions, forming intermediate metal–phytochemical complexes. This chelation process promotes homogeneous distribution of metal ions, controls nucleation and crystal growth, reduces particle agglomeration, and enhances nanoparticle stability. These naturally derived gel-like systems have attracted growing interest as sustainable alternatives to conventional synthetic media for the preparation of functional nanomaterials.

Most conventional nanoparticle synthesis methods are associated with several challenges, including instability, uncontrolled crystal growth, and particle aggregation. Additionally, the potential toxicity of nanomaterials poses significant environmental concerns. Thus, utilizing biomolecules from living plants in nanoparticle synthesis offers a promising alternative, as these biomolecules may reduce the toxicity of metallic ions by converting them into less harmful nanoscale particles. A lot of metal and metal oxide nanomaterials have been attractively gained and synthesized via green chemistry. Gold nanoparticles, a metallic nanoparticle with unique surface plasmon characteristics, have been produced using leaf, flower, and seed extraction, which have been applied in antibacterial activity and photocatalytic reactions [9]. These researchers report a new, eco-friendly, and rapid approach to fabricating metal nanoparticles, yielding functional materials for biomedical and therapeutic applications. Meanwhile, some metal oxide materials have been identified and attempted to be synthesized at the nanoscale. The fabrication of titanium dioxide (TiO_2_) using *Acorus calamus* (*A. calamus*) leaf extract as a capping and reducing agent via a green synthesis method, and the evaluation of its efficiency in photocatalytic and antimicrobial activity [10]. The biosynthesized TiO_2_ nanoparticles exhibited strong photocatalytic activity, degrading 96.59% of the RhB dye, and excellent antimicrobial activity against the selected Gram-positive bacteria in comparison to bare TiO_2_. Meanwhile, zinc oxide (ZnO) nanoparticles exhibit unique properties suitable for applications in electronic devices, antibacterial materials, and photocatalysis [11,12,13]. Furthermore, ZnO nanoparticles are non-toxic, biocompatible, and cost-effective, making them highly versatile for applications in agriculture, medicine, and environmental treatment. Additionally, metal oxide-based photocatalysts, e.g., TiO_2_ and ZnO can effectively degrade complex organic pollutants into smaller and less toxic compounds through photocatalytic oxidation processes. This degradation is primarily driven by reactive oxygen species (ROS), particularly hydroxyl radicals (•OH^−^) and superoxide radicals (•O_2_^−^), which are generated during photocatalytic reactions and serve as the main active species responsible for pollutant decomposition. Despite extensive research efforts, further enhancement of photocatalytic activity remains a critical challenge. Improving the separation and transfer efficiency of photogenerated charge carriers by material engineering or morphological decoration is considered one of the most promising approaches, as it suppresses electron–hole recombination and increases ROS generation, ultimately leading to superior photocatalytic degradation performance [14,15,16]. Algal-based zinc oxide (ZnO) nanoparticles have been fabricated by the interaction of bioactive molecules in *Spirogyra hyalina* extract with Zn^2+^ ions, demonstrating remarkable antibacterial efficacy against both Gram-positive and Gram-negative bacteria and strong antioxidant activity. The antibacterial mechanism of ZnO nanostructures primarily involves the release of Zn^2+^ ions and the generation of reactive oxygen species (ROS), both of which play crucial roles in bacterial inactivation. The generated ROS induces oxidative stress by attacking the bacterial cell wall and membrane, leading to membrane disruption, intracellular leakage, and damage to vital cellular components such as proteins, ribosomes, and DNA. Consequently, essential biological processes, including DNA replication and cellular metabolism, are severely impaired. Furthermore, owing to their nanoscale size and high surface reactivity, ZnO nanoparticles can readily diffuse onto the bacterial surface and directly interact with the cell envelope, causing physical damage to the cell wall and internal cellular structures. These combined chemical and physical antibacterial actions synergistically enhance the bactericidal efficiency of ZnO nanostructures [17]. The bioactive ZnO nanoparticle proposed in a preliminary in vitro antibacterial study has been reported by green chemistry with the aqueous extract of mangosteen leaves. Significant potential to suppress the growth of both Gram-positive and Gram-negative bacteria, with decreased minimum inhibitory concentrations, was observed with ZnO NPs [18].

In this study, ZnO nanopowders were synthesized via a green synthesis approach using *Mitragyna speciosa* Korth. leaf extracts prepared with different solvents in the extraction process. The selection of extraction solvent plays a crucial role in determining the composition and concentration of phytochemicals, owing to differences in solvent polarity, selectivity, solubility, and extraction efficiency. In addition, the influence of extraction volume, which reflects the relative phytochemical concentration available during synthesis, was also investigated on ZnO formation. Since phytochemical constituents can act as complexing, stabilizing, and capping agents during ZnO formation, variations in solvent-mediated phytochemical environments are expected to influence nucleation kinetics, crystal growth, and aggregation behavior. Consequently, variations in crystallinity, morphology, particle size, and functional performance of ZnO materials through a green synthesis route can be expected when different extraction solvents are employed. The structural and chemical properties of the synthesized ZnO nanopowders were analyzed using X-ray diffraction (XRD), scanning electron microscopy (SEM), Raman spectroscopy, and Fourier transform infrared spectroscopy (FTIR). The antibacterial activity of green-synthesized ZnO was evaluated by measuring clear zones against both Gram-positive and Gram-negative bacteria. Furthermore, the photocatalytic performance of ZnO material was assessed under piezo-assisted conditions with UV–visible light irradiation.

## 2. Results and Discussion

**Figure 1:**
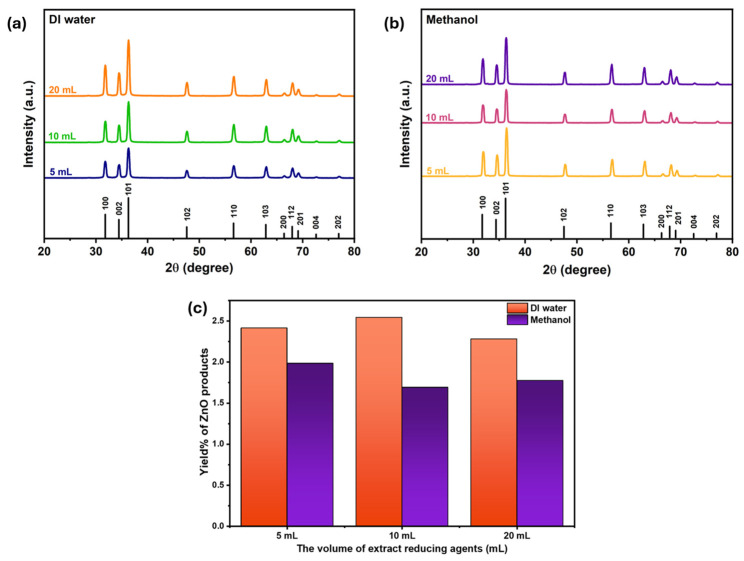
XRD patterns of ZnO nanopowders using the leaf extracts from *Mitragyna speciosa* Korth. by (**a**) DI water, (**b**) methanol, and (**c**) yield percentages of ZnO products from green synthesis.

The XRD patterns of ZnO nanopowders synthesized using different solvent extraction methods within the 2θ range of 20° to 80°, along with their yield percentages, are illustrated in Figure 1. The ZnO products synthesized from leaf extract using DI water and methanol, with calcination at 450 °C, exhibited strong crystallinity and high phase purity. The diffraction peaks observed at 2θ of 31.7°, 34.4°, 36.2°, 47.5°, and 56.5° were indexed to the crystalline planes of (100), (002), (101), (102), and (110), respectively, corresponding to a hexagonal wurtzite structure (ICSD No. 00-050-2241). The identical XRD patterns of ZnO powders remained consistent, regardless of the extraction solvent, with increasing leaf extract concentration leading to enhanced crystallinity, as depicted in Figure 1a,b. At low concentrations, biomolecules function as mild stabilizers, selectively absorbing onto high-energy facets and facilitating near-equilibrium crystal growth [19]. Thus, a higher phytochemical concentration promotes controlled nucleation and crystal growth while acting as a capping agent, leading to enhanced crystallite ordering, minimized structural defects, and stabilized crystal facets, thereby improving overall crystallinity. The formation of ZnO nanoparticles is primarily attributed to the hydrolysis of zinc acetate dihydrate in a medium, leading to the generation of Zn^2+^ ions, as proposed in Equation (1). In the environment of *Mitragyna speciosa* leaf extract, bioactive phytochemicals, including flavonoids, polyphenols, alkaloids, tannins, and proteins, interact with Zn^2+^ ions derived from zinc acetate precursor to form Zn^2+^–Phytochemical coordination complex denoted as [Zn–Phytochemical]^2+^. These phytochemicals not only act as chelating and stabilizing agents but also form a transient gel-like biomolecular network surrounding Zn^2+^ ions. This soft gel matrix restricts the rapid diffusion of precursor species, moderates nucleation kinetics, and promotes homogeneous crystal growth before calcination removes the organic matrix, as described in Equation (2). Subsequently, hydroxyl ions (OH^¯^) generated during the hydrolysis by phytochemicals and the solvent interact with free Zn^2+^ ions or [Zn–Phytochemical]^2+^ complexes, leading to the formation of Zn(OH)_2_ intermediate, as shown in Equation (3). Simultaneously, residual phytochemicals adsorb onto the Zn(OH)_2_ surface through hydroxyl, carbonyl, and carboxyl functional groups, forming a protective layer that acts as a capping and stabilizing agent. This surface passivation helps regulate particle growth, control morphology, and prevent agglomeration. Finally, thermal treatment (calcination process) induces the dehydration of Zn(OH)_2_ intermediate and removes residual organic constituents, resulting in the formation of crystalline ZnO nanopowders, as mentioned in Equation (4) [20]. The overall mechanism for the green synthesized ZnO nanoparticles using *Mitragyna speciosa* leaf extract is summarized in Equations (1–4);

Equation (1): Hydrolysis of zinc precursor:

(1) $${\;{Zn({CH}_{3}COO)}_{2}\cdot {2H}_{2}O}_{\left(s\right)}\;\overset{Hydrolysis}{\to }{Zn}_{\left(aq\right)}^{2+}+{{2CH}_{3}COO}_{\left(aq\right)}^{-}$$

Equation (2): Formation of Zn–phytochemical complexes:

(2) $${Zn}_{\left(aq\right)}^{2+}+Phytochemicals\to {\left[Zn-Phytochemical\right]}^{2+}$$

Equation (3): Formation of zinc hydroxide intermediates:

(3) $${\left[Zn-Phytichemical\right]}^{2+}+2{OH}^{-}\to {Zn(OH)}_{2\left(s\right)}+Phytochemical$$

Equation (4): Thermal decomposition

(4) $${Zn\left(OH\right)}_{2(s)}\overset{∆}{\to }{ZnO}_{\left(s\right)}+{H_{2}O}_{\left(v\right)}$$

Furthermore, the yield percentages for all ZnO products in Figure 1c range from 50% to 70%, calculated using Equation (5);

(5) $$Yield\%\;=\frac{Actual\;yield\left(g\right)\times Molarmass\;of\;zinc\;precursor}{Mass\;of\;zinc\;precursor\times Molarmass\;of\;ZnO}$$

ZnO powder synthesized using DI water as the extraction solvent exhibited a higher yield percentage compared to ZnO obtained from methanol extraction. This behavior can be attributed to the faster hydrolysis of zinc acetate in aqueous media, which promotes rapid nucleation and simultaneous growth. Thus, increased ZnO formation can be obtained by water-based extraction. In contrast, ZnO yield in the methanol system with lower polarity leads to slower hydrolysis kinetics and promotes more controlled nucleation, resulting in reduced product yield. Then, the critical role of solvent polarity in governing ZnO formation efficiency.

**Figure 2:**
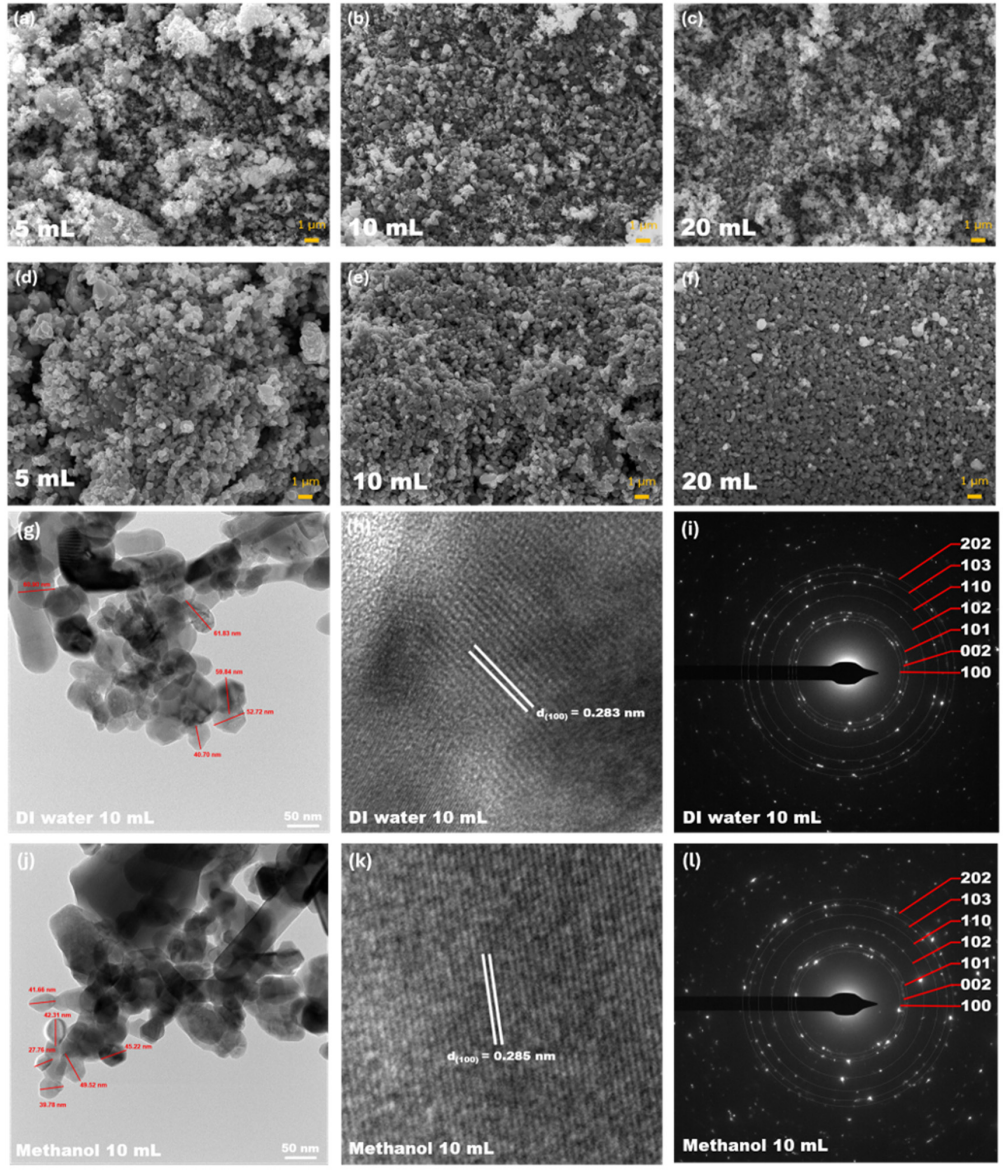
FE-SEM images of ZnO nanopowders synthesized using *Mitragyna speciosa* Korth. leaf extracts prepared with (**a–c**) deionized (DI) water and (**d–f**) methanol as extraction solvents. Brigth-field, high-resolution TEM, and selected area electron diffraction TEM images of ZnO nanopowders synthesized using (**g–i**) DI water and (**j–l**) methanol leaf extracts with different extraction solution volumes (5, 10, and 20 mL).

The surface morphologies of green-synthesized ZnO powders prepared with various media and concentrations are monitored using field-emission scanning electron microscopy, as illustrated in Figure 2. The structures of all ZnO samples are similar in spherical shapes. However, diverse morphologies observed in zinc oxide products with DI water extraction occurred in flake-like structures, ultrafine particles, and large aggregation, attributed to variations in growth rates due to high polarity and fast hydrolysis. The reaction in the growth of the crystal nucleus is subjected to less confinement in boiling droplets of solvent. Therefore, the mixed phases of rectangular and trigonal morphology in water extract conditions [21]. The heterogeneous morphologies and larger particle sizes are attributed to rapid nucleation and growth, insufficient stabilization, and the presence of diverse biomolecular species. The average particle sizes of ZnO samples with the DI water extract concentration of 5, 10, and 20 mL were approximately 458, 339, and 310 nm, as observed in Figure 2a–c. Meanwhile, homogeneous and spherical ZnO nanoparticles were clearly obtained via leaf extraction in methanol. The average particle sizes of ZnO samples with the methanol extract concentration of 5, 10, and 20 mL were approximately 338, 303, and 296 nm, as shown in Figure 2d–f. The lower particle size and spherical ZnO morphology obtained with methanol extraction indicate that the organic solvent plays a key role in controlling nucleation and crystal orientation [21]. Meanwhile, a significant decrease in particle size of green-synthesized ZnO was observed, resulting from increased leaf extract concentrations. At a low concentration of 5 mL, an insufficient number of biomolecules in the extract may be sufficient to trigger the reduction step, due to rapid depletion by metallic ions, particle aggregation, and the formation of larger structures [22]. Meanwhile, the reduction of ZnO particles could be minimized by increasing the concentration of the reducing agent to 10 and 20 mL, owing to sufficient electron donors from the phytochemicals to provide ultrafine particles. Moreover, excessively concentrated biological extracts in the green synthesis method can accelerate particle growth, leading to the formation of larger NPs [23]. To further verify the morphology and crystalline characteristics of the green-synthesized ZnO, TEM analyses are performed on representative samples synthesized using 10 mL of DI water and methanol extracts, as shown in Figure 2g–l. The TEM images (Figure 2g,j) reveal that both samples consist of aggregated ZnO nanoparticles with irregular and spherical morphologies. Consistent with the FE-SEM observations, the methanol-derived ZnO exhibits smaller and more uniformly distributed particles with reduced agglomeration compared with the DI-water-derived sample. The HRTEM images (Figure 2h,k) display well-resolved lattice fringes with interplanar spacings of approximately 0.283 and 0.285 nm for the DI-water- and methanol-derived ZnO, respectively. These lattice spacings are consistent with the (100) crystallographic plane of hexagonal wurtzite ZnO, confirming the high crystallinity of the synthesized products. Furthermore, the selected-area electron diffraction (SAED) patterns shown in Figure 2i,l exhibit a series of concentric diffraction rings indexed to the (100), (002), (101), (102), (110), (103), and (202) planes of hexagonal wurtzite ZnO. The well-defined diffraction rings demonstrate the polycrystalline nature of both samples and are in excellent agreement with the XRD results.

**Figure 3:**
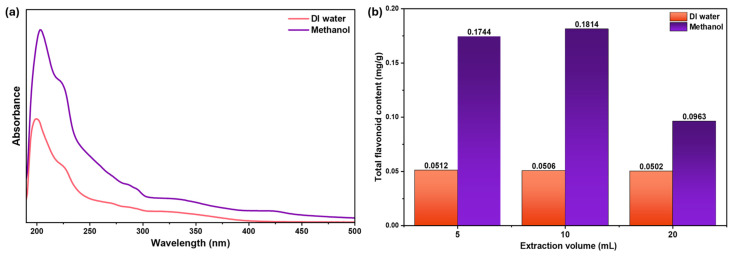
(**a**) UV-Vis absorbance spectra and (**b**) total flavonoid contents of the leaf extracts from *Mitragyna speciosa* Korth. using DI water and methanol as extraction solvents.

To describe the formation of spherical ZnO nanopowders synthesized using the methanolic leaf extract, absorbance spectra and total flavonoid contents of the leaf extracts by DI water and methanol are further investigated, as shown in Figure 3. Both extracts exhibit broad absorption bands spanning the ultraviolet and visible regions (Figure 3a), confirming the presence of diverse phytochemicals in the leaf extracts. The absorption band at 200–220 nm is associated with fundamental biomolecules and aromatic organic compounds, attributing to proteins, amino acids, and certain complex organic molecules. These biomolecular constituents indicate that the extract contains functional molecules capable of participating in the green synthesis of ZnO nanomaterials. A second absorption band at 250–280 nm corresponds to phytochemicals belonging to the phenolic acid and alkaloid groups, particularly the alkaloid mitragynine, which are among the major constituents of *Mitragyna speciosa* leaves. The existence of hydroxyl (–OH) groups and other electron-donating functional groups, indicated in this absorption band, facilitates the reduction of Zn^2+^ ions during the synthesis process. Furthermore, an absorption band was observed at approximately 300–350 nm, characteristic of flavonoids and phenolic acids. These phytochemicals function as reducing, capping, and stabilizing agents during the green synthesis process, promoting the reduction of zinc ions, controlling nucleation and crystal growth, and preventing the agglomeration of ZnO nanoparticles [24]. The extraction solvent significantly influenced the phytochemical composition of *Mitragyna speciosa* leaf extract, resulting in a biomolecular network with gel-like characteristics during green synthesis and stabilization of ZnO nanoparticles. Water extraction yielded a greater amount of extract, likely due to the efficient extraction of abundant hydrophilic constituents such as polysaccharides, proteins, and sugars. In contrast, methanol extraction produced extracts with higher flavonoid content. Flavonoids and other phenolic compounds can strongly adsorb onto ZnO crystal surfaces and act as capping agents, leading to more isotropic crystal growth and the formation of spherical nanoparticles. These results correspond to the total flavonoid contents, as reported in Figure 3b. The methanolic extract exhibits a stronger absorption intensity in the 300–350 nm region than the DI water extract, indicating a higher concentration of flavonoids. The greater abundance of flavonoids in the methanolic extract provides more effective surface capping and growth regulation, suppressing anisotropic crystal growth, and leading to the formation of more uniform spherical ZnO nanoparticles. In contrast, the lower flavonoid content in the DI water extract provides weaker growth control, allowing preferential crystal growth along the c-axis and resulting in the characteristic hexagonal ZnO morphology.

**Figure 4:**
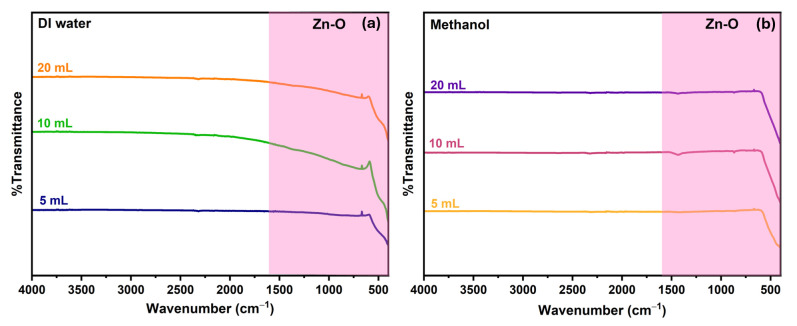
FTIR spectra of ZnO nanopowders using the leaf extracts from *Mitragyna speciosa* Korth. by (**a**) DI water and (**b**) methanol.

The FTIR spectra of ZnO nanopowders synthesized using different extraction media and concentrations are presented in Figure 4. All samples exhibited well-defined absorption bands in the range of 400–600 cm^−1^ and similar patterns corresponding to the characteristic functional group of Zn–O stretching vibration [25]. This result confirms that the crystalline ZnO framework is preserved, regardless of the extraction solvent, via the green synthesis method using *Mitragyna speciosa* Korth. leaf, according to the XRD discussion. Additionally, no characteristic absorption bands corresponding to organic functional groups (e.g., C–O, C=O, and O–H stretching vibrations) were observed, indicating the absence of residual phytochemicals from the green synthesis process. This result suggests that organic residues derived from *Mitragyna speciosa* extract were effectively removed during calcination. Furthermore, the disappearance of hydroxyl-related features supports the complete conversion of the intermediate Zn(OH)_2_ phase into ZnO. Therefore, the role of plant-derived biomolecules from both DI water and methanol extractions is primarily confined to the initial stages of the synthesis process, where they promote nucleation and act as reducing, capping, and stabilizing agents without being retained in the final ZnO structure. During synthesis, these phytochemical constituents provide a transient biomolecular network through intermolecular interactions as observed in natural gel systems. Although this organic network is removed during calcination, it plays an important role in nucleation behavior and particle growth, thereby determining the final morphology of the ZnO product. Consequently, despite the similar FTIR profiles of the final products, differences in morphology, particle size, and crystallinity indicate that the extraction solvent influences ZnO formation through solvent-dependent phytochemical environments, thereby improving photocatalytic performance.

**Figure 5:**
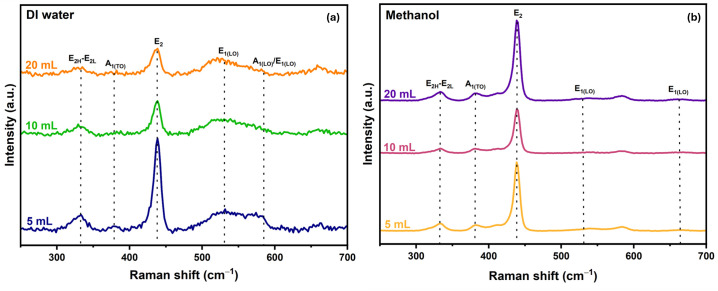
Raman spectra of ZnO nanopowders using the leaf extracts from *Mitragyna speciosa* Korth. by (**a**) DI water and (**b**) methanol.

The Raman spectra of ZnO nanopowder with various concentrations of deionized water and methanol are illustrated in Figure 5. The results reveal similar spectra across the different concentrations of extracted reducing agents, whether in deionized water or methanol. The observed Raman spectra in the ZnO powder correspond to the Raman-active vibrational modes of the wurtzite structure, with no impurity-related peaks evident in the Raman spectrum corresponding to XRD patterns in Figure 1 [26]. According to group theory, the active Raman modes of wurtzite ZnO are described as *A*_1_ + *E*_1_ + 2*E*_2_. The ZnO powder exhibited peaks around 381, 437, 530, and 585 cm^−1^, associated with one transverse *A*_1_ mode, two 2*_E_* vibrations, one longitudinal *E*_1_ mode, and *A*_1_*_(LO)_* */ E*_1_*_(LO)_* for single crystalline ZnO materials [27]. Meanwhile, the weak and broad Raman band centered at 323 cm^−1^ can be attributed to the second-order Raman scattering mode associated with zone boundary phonons of the hexagonal wurtzite ZnO structure [28]. The highest peak from the 2*E*_2_ vibrations is attributed to the nonpolar optical phonons *E_2H_*, associated with the vibration of the oxygen sublattice in the wurtzite ZnO lattice. The presence of a weaker peak corresponding to the transverse optical *A*_1_*_(TO)_* mode and the intensity of the *E_2H_* mode indicate good crystalline quality of the ZnO, while the *E_2L_* mode is due to vibrations of the heavier Zn sublattice [29]. Additionally, the *E_1(LO)_* mode is attributed to second-order Raman scattering, and a multi-phonon scattering band is observed at ≈664 cm^−1^ [30]. The concentration-dependent peak shifts and/or broadening observed in the Raman spectra can be correlated with increased disorder in the crystals induced by the presence of extract molecules. Therefore, the Raman and FT-IR results reflect the chemical bonding characteristics, aligning with the chemical fingerprint identified in pure ZnO powder.

**Figure 6:**
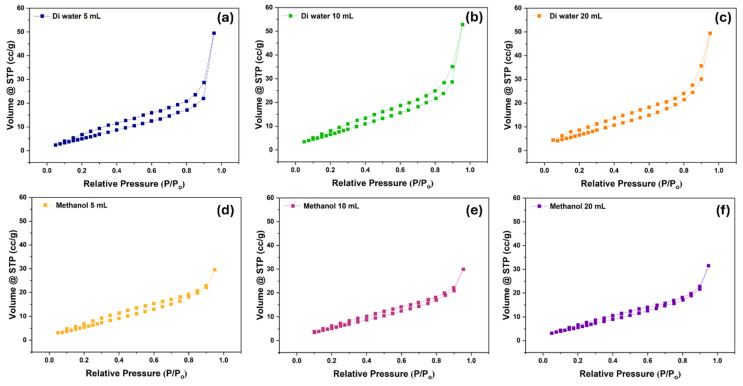
N_2_ adsorption–desorption isotherms of ZnO nanopowders synthesized using *Mitragyna speciosa* leaf extracts with (**a**–**c**) DI water and (**d**–**f**) methanol at varying extract concentrations.

The nitrogen adsorption/desorption isotherms of the ZnO nanopowders, obtained by the Brunauer–Emmett–Teller (BET) technique, are presented in Figure 6. All samples synthesized with different solvent extracts and concentrations exhibited the isotherms classified as Type IV with H3 hysteresis loops, according to IUPAC criteria, indicating mesoporous structures with capillary condensation occurring within the mesopores [31]. The isotherms with low-altitude hysteresis loops were especially observed in the samples prepared with methanol extract. The corresponding values of a specific surface area, cumulative pore volume, and average pore diameter are summarized in Table 1. The average pore diameter of all ZnO nanopowders ranged from 4.03 to 9.87 nm, confirming their mesoporous nature. The sample prepared using 20 mL of DI water showed the highest specific surface area (31.04 m^2^g^–1^), attributed to its smaller average particle size and flake-like morphology. Although the methanol-derived ZnO exhibited smaller and more uniformly distributed particles according to the FE-SEM analysis, its specific surface area was slightly lower than that of the DI-water-derived ZnO. This observation suggests that the higher surface area of the latter may be associated with differences in pore formation, particle aggregation, or surface roughness rather than particle size alone. The larger particles synthesized using the DI-water extract may form a more loosely packed aggregate network with greater interparticle voids, resulting in a relatively higher accessible surface area. In contrast, the smaller methanol-derived particles tend to form more compact aggregates during calcination, leading to a slight reduction in the measured BET surface area [32].

**Figure 7:**
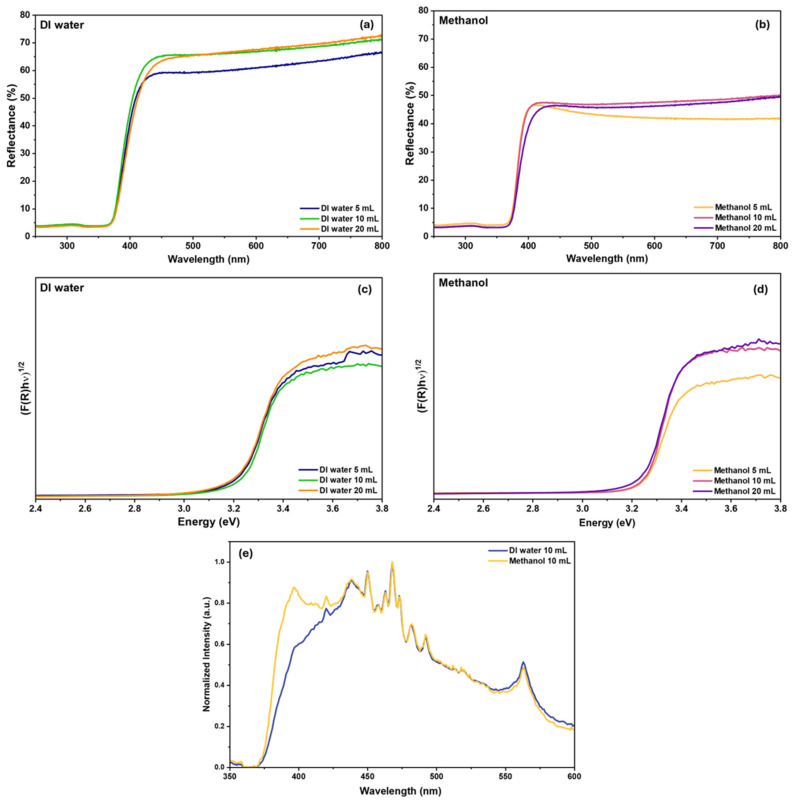
(**a**,**b**) Diffuse reflectance spectra, (**c**,**d**) optical band gap calculation, and (**e**) fluorescence spectra of ZnO nanopowders using the leaf extracts from *Mitragyna speciosa* Korth. with different solvents and extract concentrations.

Figure 7 presents the optical properties of green-synthesized ZnO nanopowders prepared using different extraction solvents and extract concentrations, as determined from diffuse reflectance and fluorescence spectra. As shown in Figure 6a,b, all samples exhibited strong UV absorption, characteristic of ZnO semiconductors synthesized via the green route and confirming their ability to activate under UV irradiation. The methanol-derived ZnO sample shows higher absorbance in the visible region than ZnO synthesized using the DI water extract. This enhanced absorption is attributed to the higher concentration of flavonoids and other phenolic compounds extracted by methanol, which readily adsorb onto ZnO crystal surfaces. The presence of these phytochemicals during the synthesis process can introduce surface states and residual organic species that contribute to increased visible-light absorption. This observation is consistent with the extraction analysis shown in Figure 3, which indicates that the methanolic extract had a significantly higher total flavonoid content than the DI water extract. The optical band gap energies in Figure 6c–d were estimated using the Kubelka–Munk function according to this equation [33].

(6) $$F\left(R\right)=\frac{{\left(1-R\right)}^{2}}{2R}$$

The calculated band gap energies ranged from 3.19 to 3.24 eV, as summarized in Table 1. ZnO synthesized using DI water extract exhibited band gap values of 3.20, 3.22, and 3.19 eV for extract concentrations of 5, 10, and 20 mL, respectively. While methanol-derived showed slightly higher values of 3.24, 3.23, and 3.22 eV with increasing extract concentration. These values are consistent with the reported band gap of hexagonal wurtzite ZnO [34]. The relatively small variation in band gap energy suggests that the extraction solvent and extract concentration did not significantly alter the intrinsic electronic structure of ZnO. This observation is consistent with the XRD, FTIR, and Raman results, which confirmed the formation of a similar ZnO crystal structure under all synthesis conditions. Furthermore, the fluorescence spectra shown in Figure 6e exhibit similar emission profiles for ZnO synthesized using 10 mL of DI water and methanol extracts, suggesting that both samples possess comparable defect-related emission characteristics. The broad visible emission around 375–600 nm is commonly attributed to deep-level intrinsic defects in ZnO, particularly oxygen vacancies, zinc interstitials, oxygen interstitials, and zinc vacancies [35]. Notably, the methanol-derived ZnO exhibited a slightly lower emission intensity than the DI-water-derived ZnO in the range of 550–600 nm, indicating a reduced defect-assisted recombination rate of photogenerated charge carriers. This behavior may contribute to the enhanced photocatalytic performance observed for the methanol-derived ZnO by facilitating more efficient charge separation and utilization during reactive oxygen species generation.

**Figure 8:**
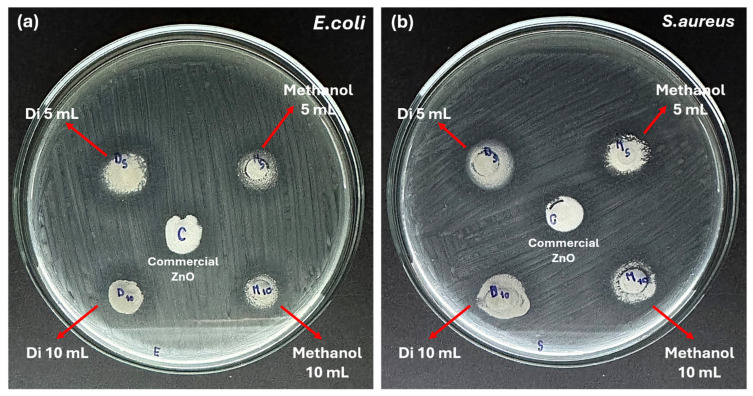
Antibacterial activity against (**a**) *E. coli* and (**b**) *S. aureus* using green-synthesized ZnO nanopowders from the leaf extracts using the different solvents of DI water and methanol.

The antibacterial activity of green-synthesized ZnO nanopowders prepared using *Mitragyna speciosa* leaf extractions is evaluated against Gram-positive *Staphylococcus aureus* (*S. aureus*) and Gram-negative *Escherichia coli* (*E. coli*) using the agar disk diffusion method, as shown in Figure 8. The mean diameters of the inhibition zone by ZnO nanopowders synthesized using different aqueous media (DI water and methanol) for leaf extraction, as well as varying extract concentrations of 5, 10, and 20 mL, were compared with those of commercial ZnO nanoparticles (control treatment), as summarized in Table 2. The highest inhibition zone diameter was observed for *E. coli* (22 ± 0.10 mm) and *S. aureus* (23 ± 0.25 mm) when ZnO nanopowders were synthesized using DI water in leaf extraction with a concentration of 5 mL. In the case of methanol extraction, the maximum inhibition zone against *E. coli* was approximately 18 ± 0.21 mm at an extract concentration of 10 mL, whereas the largest clear zone against *S. aureus* was about 23 ± 0.21 cm at 5 mL of leaf extract. Overall, effective green-synthesis ZnO powders exhibited antibacterial activity comparable to that of commercial ZnO nanoparticles, with clear zones of approximately 22 mm for both *E. coli* and *S. aureus*. The antibacterial mechanisms of ZnO nanopowders primarily arise from the release of Zn^2+^ ions, which generate reactive oxygen species (ROS) [36]. Both Zn^2+^ ions and ROS interact with the bacterial cell wall, leading to intracellular leakage and the disruption of essential biomolecules such as ribosomes and DNA, ultimately interfering with DNA replication [37,38]. Furthermore, the bacterial cell wall can be directly damaged by small-scale ZnO particles through diffusion on the cell surface [31], leading to internal structural damage and contributing to bacterial inhibition.

In addition, the antibacterial activity of green-synthesized ZnO nanoparticles obtained from leaf extraction using DI and methanol at 5 mL was further evaluated by determining the minimum inhibitory concentration (MIC) and minimum bactericidal concentration (MBC) against *E. coli* and *S. aureus*. As summarized in Table 3, ZnO synthesized using the 5 mL DI water extract exhibited MIC values of 6.25 and 0.39 mg mL^−1^ for *E. coli* and *S. aureus*, respectively, while the corresponding MBC values are 100 and 50 mg mL^−1^. Similarly, ZnO synthesized using the 5 mL methanolic extract showed MIC values of 6.25 and 0.39 mg mL^−1^ against *E. coli* and *S. aureus*, respectively, with MBC values of 100 and 25 mg mL^−1^. The lower MIC and MBC values obtained against *S. aureus* compared with *E. coli* indicate that the synthesized ZnO nanoparticles were more effective against Gram-positive bacteria, as evidenced by the disk diffusion assay. This behavior may be attributed to the structural differences in bacterial cell walls, where the outer lipopolysaccharide membrane of Gram-negative *E. coli* provides an additional barrier against nanoparticle penetration and reactive oxygen species (ROS)-induced damage [39]. Furthermore, ZnO synthesized using the methanolic extract exhibited slightly lower MIC and MBC values than the corresponding DI water-derived sample, suggesting enhanced antibacterial activity that may be associated with differences in particle morphology, crystallinity, surface chemistry, and the phytochemical composition retained during synthesis.

**Figure 9:**
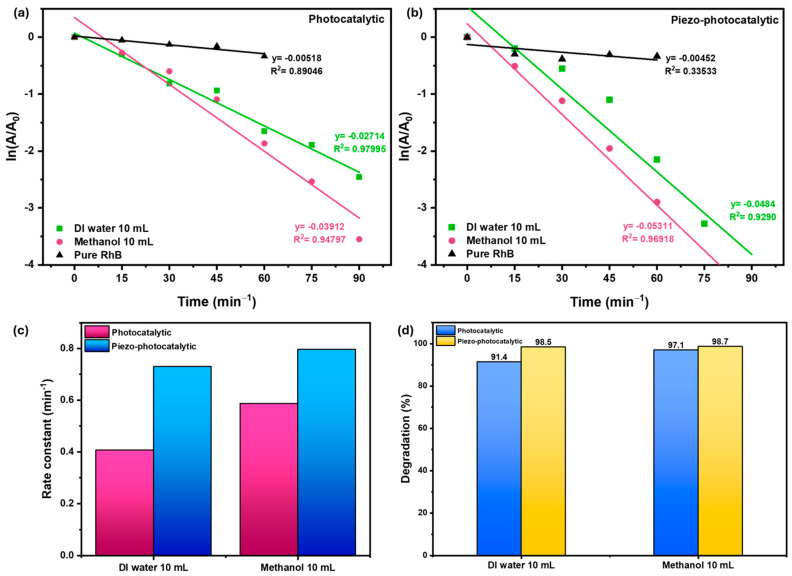
(**a**) Photocatalytic degradation, (**b**) piezo-photocatalytic degradation profiles of RhB dye, (**c**) the pseudo-first-order kinetics rate constants, and (**d**) the degradation percentage of ZnO nanopowders with 10 mL of leaf extraction by DI water and methanol.

This study investigates the efficiency of green-synthesized ZnO nanopowder in removing the organic dye Rhodamine B (RhB) via piezo-photocatalysis. The catalytic performance of ZnO nanopowder, prepared with various aqueous media of DI water and methanol in leaf extraction using an extract concentration of 10 mL, was examined under two experimental conditions: light irradiation and a combination of light and ultrasonic irradiation, as illustrated in Figure 9. The photocatalytic degradation curves for ZnO nanopowder using 10 mL of extract concentration with DI water and methanol are presented in Figure 9a. ZnO samples demonstrated the ability to degrade nearly all the RhB dye solution within 90 min. Moreover, when combined with ultrasonic irradiation in a piezo-photocatalytic setup, the degradation rate significantly increased, with nearly complete degradation occurring within 60 min, as shown in Figure 9b. Furthermore, the relationship between A/A_0_ and the degradation rate constant (*k*) follows the pseudo-first-order rate law, as depicted in Figure 9c. For the ZnO nanopowder with 10 mL of extract concentration by DI water, the piezo-photocatalytic and photocatalytic degradation rate constants (*k*) were calculated as 0.73 (R^2^ = 0.94) and 0.41 (R^2^ = 0.98), respectively. For the ZnO nanopowder with 10 mL of an extract concentration by methanol, the corresponding values were 0.80 (R^2^ = 0.97) and 0.59 (R^2^ = 0.96), indicating an improvement. These findings highlight a strong correlation between enhanced photocatalytic properties and optimal performance under ultrasonic treatment for the ZnO nanopowder sample. Therefore, ZnO nanopowder with a 10 mL of an extract concentration using methanol exhibited better piezo-photocatalytic properties compared to the ZnO nanopowder with an extraction using DI water. The highest enhancement observed under ultrasonic irradiation is supported by Raman results, which indicate that this condition produced the highest peak from the *E_2_* vibrations associated with the nonpolar modes of the oxygen sublattice in ZnO. It is well-known that oxygen vacancies predominantly form at the (100) surface of ZnO [40]. Moreover, the enhanced photocatalytic performance of methanol-derived ZnO can also be attributed to a more homogeneous phytochemical-mediated synthesis environment. This mechanism provides improved stabilization of Zn^2+^ ions and uniform crystal growth, producing nanoparticles with fewer structural defects and highly efficient charge separation. The efficiency of ZnO nanopowder in the decomposition of RhB can be explained by the hydroxyl radicals present on the surface of ZnO, which break down the RhB dye in aqueous solutions. Under light irradiation, traditional photocatalytic oxidation occurs through photoinduced charge separation, generating electrons and holes that migrate to the surface of ZnO to form superoxide (${•O}_{2}^{-}$) and hydroxyl radicals (•OH), effectively decomposing the RhB chemical structure. The photocatalytic mechanisms are proposed in these equations;

(7) $$ZnO+h\upsilon \;\to \;e_{cb}^{-}+h_{vb}^{+}$$

(8) $$e_{cb}^{-}+O_{2}\to \;{•O}_{2}^{-}$$

(9) $$h_{vb}^{+}+{OH}^{-}\to \;•OH$$

(10) $${•O}_{2}^{-}+C_{28}H_{31}CIN_{2}O_{3}\;\to Oxidation\;products$$

(11) $$•OH+C_{28}H_{31}CIN_{2}O_{3}\;\to Oxidation\;products$$

Additionally, the separation of photo-generated electron–hole pairs is improved when combined with ultrasonic irradiation, reducing the recombination rate and enhancing the photocatalytic activity of the ZnO-based photocatalyst. Zinc oxide is known to frequently experience recombination of electrons and holes with aggregation of particles, which can terminate the degradation reaction and lead to inefficient dye degradation. This inefficiency is a consequence of competitive charge recombination occurring at a high rate due to poor charge separation and transport. In piezoelectric materials like ZnO, the built-in electric field generates an internal voltage under mechanical vibrations [41]. This piezoelectric field in the piezo-catalytic process drives photogenerated electrons and holes to transfer in opposite directions, thereby reducing the recombination rate. The primary reason for the improved piezo-photocatalytic effectiveness is the band bending induced by the piezoelectric field to active oxygen radicals [34]. Then the mechanical agitation from ultrasonic waves improves the mass transfer of reactants within the solution. This enhanced agitation facilitates the movement of reactants toward the catalyst surface, thereby promoting better interactions and resulting in increased reaction rates. Moreover, ultrasonic treatment activates the photocatalyst by breaking up agglomerated particles, allowing for a more uniform distribution of the catalyst while preserving the active sites. This increased surface area provides more active sites available for photocatalytic reactions, represented by the transformation from ZnO aggregated to ZnO distributed, which is vital for achieving optimal photocatalytic performance combined piezoelectric process can be described by the following formula [42];

(12) $$ZnO\;\overset{ultrasonic}{\to }\;ZnO+V_{O}^{2+}+{2e}^{-}$$

(13) $$O_{2}+e^{-}\;\to \;{\cdot O}_{2}^{-}$$

(14) $$H_{2}O+{\cdot O}_{2}^{-}\to \;\cdot OOH+{OH}^{-}$$

(15) $$2\cdot OOH\;\to O_{2}+H_{2}O_{2}$$

(16) $$OOH+H_{2}O+e^{-}\to H_{2}O_{2\;}+{OH}^{-}$$

(17) $$H_{2}O_{2\;}+e^{-}\to \;\cdot OH+{OH}^{-}$$

Therefore, the turbulence induced by ultrasonic waves helps reduce the recombination of electron–hole pairs generated during the light absorption process, thus enhancing overall photocatalytic efficiency by the green-synthesis ZnO nanopowder.

**Figure 10:**
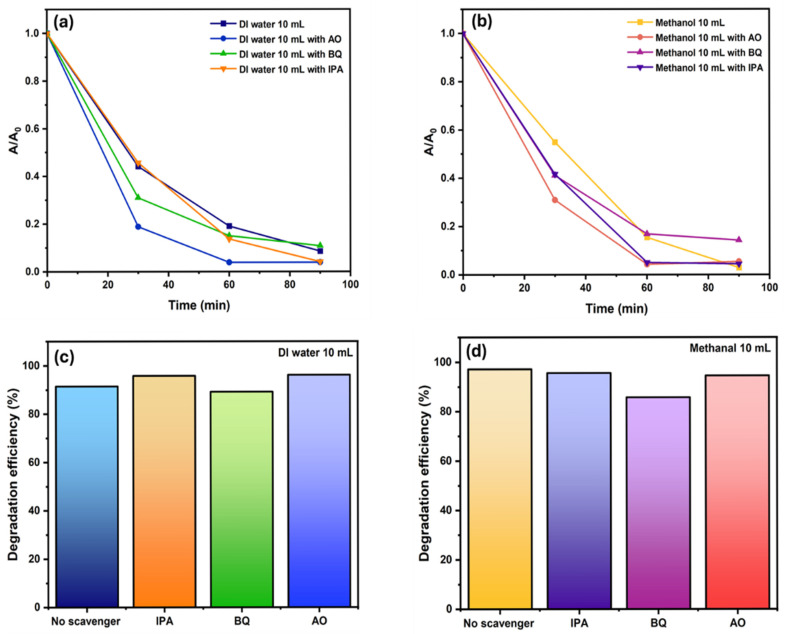
Comparative experiments with various active species scavengers for the photocatalytic degradation of RhB with (**a**,**b**) the pseudo-first-order kinetics rate constants and (**c**,**d**) the degradation efficiency by the ZnO nanopowder with 10 mL of leaf extraction by DI water and methanol.

The results of the scavenger study, which aimed to identify the primary reactive species responsible for RhB dye degradation using green-synthesized ZnO nanopowder, are presented in Figure 10. It is well known that reactive species such as photogenerated h^+^, ${•O}_{2}^{-}$, and •OH radicals play important roles in the photocatalytic process, influenced by the presence of AO, BQ, and IPA scavenger solution. The relationship between (A/A_0_) for different active species scavengers in the photocatalytic degradation curves by ZnO nanopowder using 10 mL of extract concentration with DI water and methanol is illustrated in Figure 10a,b. The trapping experiment results in Figure 10c,d indicate that RhB photodegradation rate remains highly efficient, reaching near completion within 90 min in the presence of AO and IPA, owing to the contribution of free radicals in the degradation mechanism. In contrast, the addition of BQ solution in the photocatalytic reaction significantly reduced catalytic activity, highlighting the crucial role of superoxide radicals primarily through the incorporation of conduction band electrons with oxygen molecules. Thus, the strong interaction between BQ and superoxide radicals relates to the lowest photocatalytic degradation rate. These results confirm that the green-synthesized ZnO nanopowders, prepared using different solvent extracts, primarily generate superoxide radicals; ${•O}_{2}^{-}$ as the dominant reactive species, consistent with the behavior of bare ZnO powder in chemical processes.

The extraction solvent significantly influenced the phytochemical composition of the *Mitragyna speciosa* leaf extracts and consequently affected the properties of the synthesized ZnO nanoparticles. Water extraction yielded a greater amount of extract and produced ZnO with a higher specific surface area and enhanced antibacterial activity. In contrast, methanol extraction resulted in higher flavonoid content and generated more uniform spherical ZnO nanoparticles with superior photocatalytic performance. The observed differences may be attributed to solvent-dependent variations in phytochemical composition, which can influence nucleation, crystal growth, and particle aggregation during ZnO formation. In addition, the methanol-derived ZnO exhibited stronger visible-light absorption and lower fluorescence intensity, suggesting reduced charge-carrier recombination, which may contribute to its improved photocatalytic activity. Although these correlations are consistent with the experimental observations, further studies are required to establish direct quantitative relationships between phytochemical composition, defect structure, and catalytic performance.

## 3. Conclusions

Green-synthesized ZnO nanopowders were produced using *Mitragyna speciosa* Korth. leaf extracts, with DI water and methanol as solvents. This work demonstrates that phytochemical-rich extracts not only act as natural reducing and stabilizing agents but also provide a transient biomolecular network during green synthesis. The gel-assisted molecular environment effectively regulates ZnO nucleation, crystal growth, and defect formation, resulting in improved antibacterial and piezo-photocatalytic properties. XRD patterns, FTIR analysis, and Raman spectra confirmed that ZnO crystal structures remained consistent across different solvent types in leaf extraction, with observable increases corresponding to higher plant extraction concentration. SEM images revealed variations in ZnO morphology depending on the extraction solvent. ZnO powder synthesized using DI water conditions produced a mixture of spherical and rod-like shapes with some particle aggregation, while methanol extraction resulted in homogeneous spherical ZnO. Additionally, the effective antibacterial activity of green-synthesized ZnO was demonstrated against *E. coli* and *S. aureus* under ambient conditions, with the largest inhibition zone observed for ZnO synthesized with DI water and a leaf extract concentration of 5 mL. In the piezo-photocatalytic reaction, RhB solution was completely degraded by green-synthesized ZnO photocatalysts obtained via methanol extraction. The enhanced performance was attributed to the smaller particle size compared to that of ZnO synthesized using DI water extraction. Moreover, the piezoelectric properties of ZnO material produced via green synthesis were confirmed through rapid mechanical force response under ultrasonic-assisted light irradiation. This effect was associated with the generation of an internal electric field, which served as a barrier to electron–hole pair recombination, thereby enhancing photocatalytic efficiency. The scavenger test of the green-synthesized ZnO nanopowders using different solvent extracts confirmed that superoxide radicals were primarily generated by the fast response with BQ scavenger.

These findings broaden the application of naturally derived gel systems as sustainable reaction media for engineering functional oxide nanomaterials. c These findings broaden the application of naturally derived gel systems as sustainable reaction media for engineering functional oxide nanomaterials.

## 4. Materials and Methods

### 4.1. Materials

*Mitragyna speciosa* Korth. leaves (Kratom leaves) were obtained from the area of Pathum Thani province in Thailand. The solvents in leaf extraction, deionized water, and methanol, were used in laboratory grade. Meanwhile, zinc acetate dihydrate was chosen as a zinc precursor for ZnO production and purchased from Sigma-Aldrich (St. Louis, MO, USA).

### 4.2. Preparation of Mitragyna speciosa Korth. Leaf Extract

Firstly, Kratom leaves were washed thoroughly with deionized water to remove surface dust and impurities. The washed leaves were dried in an oven at 100 °C for 12 h, then ground into a fine powder. Afterward, 2 g of the fine leaf powder was mixed with 100 mL of a solvent comprising deionized water and methanol and shaken at 245 rpm for 5 h. Once macerated, each mixture was placed in a water bath at 60 °C for 60 min, then filtered to obtain the clear extract. Finally, the filtered extract was already used as the reducing agent for ZnO nanopowder.

### 4.3. Synthesis of ZnO Nanopowder via Mitragyna speciosa Korth. Leaf Extraction

The amount of *Mitragyna speciosa* Korth. leaf extraction with DI water and methanol solvent was performed at varying volumes of 5, 10, and 20 mL, and then blended with 2 g of zinc acetate dihydrate to facilitate ZnO synthesis. The mixtures were stirred at room temperature for 60 min and then placed in a water bath at 60 °C for 60 min. Subsequently, the suspensions were dried in an oven at 100 °C for 12 h and then calcined for 2 h at 450 °C in a furnace to obtain the nanopowder.

### 4.4. Characterization

The crystalline, chemical, and morphological structures of the ZnO nanopowders were characterized using various analytical techniques. The crystalline structure of the ZnO nanopowder was characterized using X-ray diffraction (XRD; Rigaku SmartLab, Rigaku Corporation, Tokyo, Japan ). The chemical bonding structure of the ZnO powder was analyzed by Fourier-transform infrared spectroscopy (FTIR; PerkinElmer Scientific Spectrum Two, PerkinElmer, Waltham, MA, USA) and Raman spectrometer (Thermo Scientific DXR SmartRaman, Thermo Fisher Scientific, Waltham, MA, USA). Surface morphologies of the ZnO nanopowder were monitored using a field emission-scanning electron microscope (FE-SEM; JEOL JSM-7610F, JEOL Ltd., Tokyo, Japan) and transmission electron microscopy (TEM; Thermo Scientific Talos F200X, Thermo Fisher Scientific, Waltham, MA, USA). Optical property was investigated by a diffuse reflectance spectrophotometer (HITACHI UH4150, Hitachi High-Tech Corporation, Ibaraki, Japan) while fluorescence was observed by a fluorescence spectrometer (FluoroMax+ SpectroFluorometer, Horiba Scientific (Horiba Ltd.), Kyoto, Japan). Additionally, the specific surface area per volume of the ZnO samples was analyzed using the Brunauer–Emmett–Teller (BET) method with a Quantachrome Autosorb iQ-C-XR-XR-XR instrument (Anton Paar ltd., Albans, Hertfordshire, UK).

### 4.5. Antibacterial Activity

Antibacterial activity was against using Gram-positive Staphylococcus aureus (*S. aureus*) and Gram-negative Escherichia coli (*E. coli*). These bacterial strains were cultured on Mueller–Hinton agar plates at 37 °C for 24 h. Subsequently, bacterial suspensions were prepared by direct colony suspension in 0.85% (w/v) sterile normal saline solution and adjusted to an optical density (OD) of 0.1 at 600 nm. For the agar diffusion assay, ZnO nanoparticle suspensions dispersed in DI water were deposited onto nutrient agar (NA) plates previously inoculated with the test bacteria. Commercial ZnO nanoparticles were used as the control condition. The plates were incubated at 37 °C for 24 h, after which the diameters of the inhibition zone were measured to evaluate the antibacterial efficacy of the green-synthesized ZnO nanopowder. The minimum inhibitory concentration (MIC) and minimum bactericidal concentration (MBC) were determined according to the Clinical and Laboratory Standards Institute (CLSI, 2012) guidelines with slight modifications for antimicrobial susceptibility testing of aerobic bacteria [43]. A stock ZnO nanoparticle suspension at double the desired concentration was prepared and subjected to two-fold serial dilutions in a 96-well microtiter plate, yielding final concentrations in the range of 200.00 to 0.39 mg/mL. Subsequently, 50 mL of the pathogens were added to each well containing a different concentration of ZnO suspension and incubated at 37 °C for 18 h. After that, 30 μL of 0.02% resazurin solution was added to each well, and the color change was compared with that of the growth control and sterility control wells. The lowest concentration inhibiting visual growth was considered the MIC. For MBC determination, 10 μL of the suspension was spread onto nutrient agar plate and incubated at 37 °C, overnight. The MBC was defined as the lowest concentration at which no bacterial colonies on the plate.

### 4.6. Piezo-Photocatalytic Degradation

The piezo-photocatalytic activity of green-synthesis ZnO nanopowders with various solvents of deionized water and methanol in leaf extraction was evaluated using Rhodamine B (RhB) as a model of an organic dye pollutant. A 10 µM RhB solution was tested in photocatalytic degradation under two experimental conditions: exposure to a Xenon light source (300 W, Sciencetech Inc., London, ON, Canada) and a combination of light and ultrasonic irradiation from an ultrasonic bath (70 W, 40 kHz). In both conditions, 50 mL of the RhB solution was combined with 0.1 g of ZnO nanopowder (in condition, 10 mL of reducing agent from DI water and methanol in the leaf extraction). The suspensions were stirred in the dark for 30 min to establish adsorption/desorption equilibrium on the photocatalyst surface. After that, the suspensions were exposed under Xenon irradiation (300 W) to test the photocatalytic reaction. For the piezo-photocatalytic procedure, the mixture was exposed to both a Xenon lamp and ultrasound irradiation. All samples were collected in 15 min intervals for a total of 90 min and centrifuged at 5000 rpm for 10 min. For characterization, the RhB solution without the photocatalyst after the photocatalytic test was monitored using a UV–Vis spectrophotometer (Thermo Spectronic Helios Alpha 9423 UVA 1002E) in absorbance mode. The degradation rate of the ZnO catalysts is represented in the relationship between ln (A/A_0_) and irradiation time, calculated based on the following equation [44]:

(18) $$ln\left(\frac{A}{A_{0}}\right)=-kt$$

where A is the RhB absorbance at any given time, A_0_ is the initial RhB absorbance (before testing), *k* is the reaction rate constant (min^−1^), and t is the irradiation time (minutes). The scavenger test for green-synthesized ZnO nanopowders under photocatalytic reaction was conducted using 1 mM solutions of ammonium oxalate (AO), benzoquinone (BQ), and isopropanol (IPA) as scavengers for hole (h^+^), superoxide (${\cdot O}_{2}^{-}$), and hydroxyl radicals (•OH), respectively [45]. Each scavenger solution (1 mL) was added to 25 mL of RhB solution and then exposed to a Xenon lamp for 90 min. The samples were collected every 30 min to analyze RhB absorbance using UV–Vis spectrophotometer (Thermo Spectronic Helios Alpha 9423 UVA 1002E).

## Figures and Tables

**Table 1:** Surface area per volume, cumulative volume, average pore diameter, and band gap calculation of ZnO nanopowders with different solvents and extract concentrations.

Green-ZnO Samples		BET Surface Area (m^2^ g^–1^)	Cumulative Volume (1–50 nm, cc^3^ g^–1^)	Average Pore Diameter (nm)	Band Gap Energy (eV)
Extraction Solvents	Extract Concentrations (mL)				
DI water	5	26.33	0.027	4.03	3.20
	10	28.96	0.034	4.65	3.22
	20	31.04	0.077	9.87	3.19
Methanol	5	27.17	0.046	6.74	3.24
	10	24.88	0.046	7.45	3.23
	20	26.68	0.049	7.33	3.22

**Table 2:** Clear zone measurement by green-synthesized ZnO nanopowders against *E. coli* and *S. aureus.*

Green-ZnO Samples		Zone of Inhibition (mm)	
Extraction Solvents	Extract Concentrations (mL)	*E. coli*	*S. aureus*
DI water	5	22 ± 0.10	23 ± 0.25
	10	19 ± 0.18	20 ± 0.36
	20	19 ± 0.10	20 ± 0.32
Methanol	5	17 ± 0.05	23 ± 0.21
	10	18 ± 0.21	21 ± 0.28
	20	17 ± 0.11	21 ± 0.21
Commercial ZnO		22 ± 0.15	21 ± 0.11

**Table 3:** MIC and MBC values of green-synthesized ZnO nanopowders from the leaf extracts with DI water and methanol against bacteria.

Green-ZnO Samples	MIC Value (mg mL^−1^)		MBC Value (mg mL^−1^)	
	*E. coli*	*S. aureus*	*E. coli*	*S. aureus*
DI water 5 mL	6.25	0.39	100	50
Methanol 5 mL	6.25	0.39	100	25

## Data Availability

Data are contained within the article.

## References

[1] Bhattacharjee N., Som I., Saha R., Mondal S. (2022). A Critical Review on Novel Eco-friendly Green Approach to Synthesize Zinc Oxide Nanoparticles for Photocatalytic Degradation of Water Pollutants. Int. J. Environ. Anal. Chem..

[2] Miu B.A., Dinischiotu A. (2022). New Green Approaches in Nanoparticles Synthesis: An Overview. Molecules.

[3] Kawee-Ai A. (2025). Advancing Gel Systems with Natural Extracts: Antioxidant, Antimicrobial Applications, and Sustainable Innovations. Gels.

[4] Chandrababu P., Cheriyan S., Raghavan R. (2019). Aloe Vera Leaf Extract-assisted Facile Green Synthesis of Amorphous Fe_2_O_3_ for Catalytic Thermal Decomposition of Ammonium Perchlorate. J. Therm. Anal. Calorim..

[5] Sumitha S., Vasanthi S., Shalini S., Chinni S.V., Gopinath S.C.B., Anbu P., Bahari M.B., Harish R., Kathiresan S., Ravichandran V. (2018). Phyto-Mediated Photo Catalysed Green Synthesis of Silver Nanoparticles Using *Durio zibethinus* Seed Extract: Antimicrobial and Cytotoxic Activity and Photocatalytic Applications. Molecules.

[6] Naiel B., Fawzy M., Halmy M.W.A., Mahmoud A.E.D. (2022). Green Synthesis of Zinc Oxide Nanoparticles Using Sea Lavender (*Limonium pruinosum* L. Chaz.) Extract: Characterization, Evaluation of Anti-Skin Cancer, Antimicrobial and Antioxidant Potentials. Sci. Rep..

[7] Meireles V., Rosado T., Barroso M., Soares S., Gonҫalves J., Luís Â., Caramelo D., Simão A.Y., Fernández N., Duarte A.P. (2019). *Mitragyna speciosa*: Clinical, Toxicological Aspects and Analysis in Biological and Non-biological Samples. Medicines.

[8] Veeramohan R., Zamani A.I., Azizan K.A., Goh H.H., Aizat W.M., Razak M.F.A., Yusof N.S.M., Mansor S.M., Baharum S.N., Ng C.L. (2023). Comparative Metabolomics Analysis Reveals Alkaloid Repertoires in Young and Mature *Mitragyna speciosa* (Korth.) Havil. Leaves. PLoS ONE.

[9] Boruah J.S., Devi C., Hazarika U., Reddy P.V.B., Chowdhury D., Barthakur M., Kalita P. (2021). Green Synthesis of Gold Nanoparticles Using an Antiepileptic Plant Extract: *In Vitro* Biological and Photo-Catalytic Activities. RSC Adv..

[10] Ansari A., Siddiqui V.U., Rehman W.U., Akram M.K., Siddiqi W.A., Alosaimi A.M., Hussein M.A., Rafatullah M. (2022). Green Synthesis of TiO_2_ Nanoparticles Using *Acorus calamus* Leaf Extract and Evaluating Its Photocatalytic and In Vitro Antimicrobial Activity. Catalysts.

[11] Morales-Salvador R., Demiroglu I., Viñes F., Bromley S.T. (2025). Tuning the Electronic Properties Of ZnO Nanofilms *Via* Strain-Induced Structural Phase Transformations and Quantum Confinement. Nanoscale.

[12] Athamneh T., Abuawad A., Odat T., Alshweiat A., Obaidat R., Yaseen F.B., Al-Najjar M.A., Garafat R., Altarabeen R., Smirnova I. (2025). Investigation of the Antibacterial Activity of ZnO-Loaded Alginate/Hyaluronic Acid Aerogels for Wound Dressing Applications. Polymers.

[13] Masuleh M.T., Hasheminiasari M., Ashiri R. (2025). Enhanced Photocatalytic Efficiency of Eco-friendly Synthesized ZnO for Rapid Full Degradation of Methylene Blue Dye. Mater. Adv..

[14] Maijan P., Waen-ngoen T., Suwanboon S., Chantarak S., Voravuthikunchai S.P. (2024). Sustainable Environmental-Based Synthesis of Zinc Oxide Nanoparticles Using Para Rubber Leaf Extract for Photocatalytic Degradation of Organic Pollutants and Microbial Control in Wastewater Treatment. Inorg. Chem. Commun..

[15] Yi S., Yu L., Liu L., Fan Z., Zhou Y., Zhao H., Li F., Feng X., Wang H. (2026). Coupling a Ti/SnO_2_-Sb@TiO_2_/SnO_2_ Photoanode with Ce-cu/BC Particle Electrodes for Synergistic Ofloxacin Detoxification: Insights into Mechanisms and Transformations. Chem. Eng. J..

[16] Liang S., Feng X., Li X., Wang Z., Miao K., Feng G., Luo X. (2026). Catalysis of Nonstoichiometric Mn–Co Spinel Oxides in Effective Peroxymonosulfate Activation: Structure–activity Relationship and Flow-through Organic Wastewater Treatment. Sep. Purif. Technol..

[17] Hameed H., Waheed A., Sharif M.S., Saleem M., Afreen A., Tariq M., Kamal A., Al-onazi W.A., Farraj D.A.A., Ahmad S. (2023). Green Synthesis of Zinc Oxide (ZnO) Nanoparticles from Green Algae and Their Assessment in Various Biological Applications. Micromachines.

[18] Chan Y.B., Aminuzzaman M., Rahman M.K., Win Y.F., Sultana S., Cheah S.-Y., Watanabe A., Wong L.S., Guha S.K., Djearamane S. (2024). Green Synthesis of ZnO Nanoparticles Using the Mangosteen (*Garcinia mangostana* L.) Leaf Extract: Comparative Preliminary In Vitro Antibacterial Study. Green Process. Synth..

[19] Jiménez-Rosado M., Gomez-Zavaglia A., Guerrero A., Romero A. (2022). Green Synthesis of ZnO Nanoparticles Using Polyphenol Extracts From Pepper Waste (*Capsicum annuum*). J. Clean. Prod..

[20] Kanade K.G., Kale B.B., Aiyer R.C., Das B.K. (2006). Effect of Solvents on the Synthesis of Nano-size Zinc oxide and Its Properties. Mater. Res. Bull..

[21] Yuan C.-G., Huo C., Yu S., Gui B. (2017). Biosynthesis of Gold Nanoparticles Using *Capsicum Annuum* Var. *Grossum* Pulp Extract and Its Catalytic Activity. Phys. E.

[22] Kumari M., Mishra A., Pandey S., Singh S.P., Chaudhry V., Mudiam M.K.R., Shukla S., Kakkar P., Nautiyal C.S. (2016). Physico-chemical Condition Optimization During Biosynthesis Lead to Development of Improved and Catalytically Efficient Gold Nano Particles. Sci. Rep..

[23] Nagaraju G., Udayabhanu, Shivaraj, Prashanth S.A., Shastri M., Yathish K.V., Anupama C., Rangappa D. (2017). Electrochemical Heavy Metal Detection, Photocatalytic, Photoluminescence, Biodiesel Production and Antibacterial Activities of Ag–ZnO Nanomaterial. Mater. Res. Bull..

[24] Liu J., Yong H., Yao X., Hu H., Yun D., Xiao L. (2019). Recent Advances in Phenolic-protein Conjugates: Synthesis, Characterization, Biological Activities and Potential Applications. RSC Adv..

[25] Gatou M.A., Lagopati N., Vagena I.-A., Gazouli M., Pavlatou E.A. (2022). ZnO Nanoparticles From Different Precursors and Their Photocatalytic Potential for Biomedical Use. Nanomaterials.

[26] Sarkar T., Kundu S., Ghorai G., Sahoo P.K., Bhattacharjee A. (2023). Structural, Spectroscopic and Morphology Studies on Green Synthesized ZnO Nanoparticles. Adv. Nat. Sci. Nanosci. Nanotechnol..

[27] Shah J., Kotnala R.K. (2017). Rapid Green Synthesis of ZnO Nanoparticles Using a Hydroelectric Cell Without an Electrolyte. J. Phys. Chem. Solids.

[28] Mohammad A.M., Ahmed Al-Jaf H.S., Ahmed H.S., Mohammed M.M., Khodair Z.T. (2022). Structural and Morphological Studies of ZnO Nanostructures. J. Ovonic Res..

[29] Songpanit M., Boonyarattanakalin K., Pecharapa W., Mekprasart W. (2024). ZnO Nanostructures Synthesized by One-step Sol-gel Process Using Different Zinc Precursors. J. Met. Mater. Miner..

[30] Silambarasan M., Saravanan S., Soga T. (2015). Raman and Photoluminescence Studies of Ag and Fe-Doped ZnO Nanoparticles. Int. J. ChemTech Res..

[31] Su X., Zhao X., Cui C., Xi N., Zhang X.L., Liu H., Yu X., Sang Y. (2022). Influence of Wurtzite ZnO Morphology on Piezophototronic Effect in Photocatalysis. Catalysts.

[32] Godoy-Gallardo M., Eckhard U., Delgado L.M., Puente Y.J.D.D.R., Hoyos-Nogués M., Gil F.J., Perez R.A. (2021). Antibacterial Approaches in Tissue Engineering Using Metal Ions and Nanoparticles: From Mechanisms To Applications. Bioact. Mater..

[33] Makuła P., Pacia M., Macyk W. (2018). How To Correctly Determine the Band Gap Energy of Modified Semiconductor Photocatalysts Based on UV–Vis Spectra. J. Phys. Chem. Lett..

[34] Pal U., Kim C.W., Jadhav N.A., Kang Y.S. (2009). Ultrasound-Assisted Synthesis of Mesoporous ZnO Nanostructures of Different Porosities. J. Phys. Chem. C.

[35] Catto A.C., da Silva L.F., Bernardi M.I.B., Li M.S., Longo E., Lisboa-Filho P.N., Nascimento O.R., Mastelaro V.R. (2014). An Investigation into The Influence Of Zinc Precursor on the Microstructural, Photoluminescence, and Gas-Sensing Properties Of ZnO Nanoparticles. J. Nanopart. Res..

[36] Al-Askar A.A., Hashem A.H., Elhussieny N.I., Saied E. (2023). Green Biosynthesis of Zinc Oxide Nanoparticles Using *Pluchea Indica* Leaf Extract: Antimicrobial and Photocatalytic Activities. Molecules.

[37] Vitasovic T., Caniglia G., Eghtesadi N., Ceccato M., Bøjesen E.D., Gosewinkel U., Neusser G., Rupp U., Walther P., Kranz C. (2024). Antibacterial Action of Zn^2+^ Ions Driven by the In Vivo Formed ZnO Nanoparticles. ACS Appl. Mater. Interfaces.

[38] Ijaz M., Zafar M., Islam A., Afsheen S., Iqbal T. (2020). A Review on Antibacterial Properties of Biologically Synthesized Zinc Oxide Nanostructures. J. Inorg. Organomet. Polym. Mater..

[39] Zarrindokht E.K., Pegah C. (2012). Antibacterial activity of ZnO nanoparticle on Gram-positive and Gram-negative bacteria. Afr. J. Microbiol. Res..

[40] Chimupala Y., Phromma C., Yimklan S., Semakul N., Ruankham P. (2020). Dye Wastewater Treatment Enabled by Piezo-Enhanced Photocatalysis of Single-Component ZnO Nanoparticles. RSC Adv..

[41] Jing L., Xu Y., Xie M., Li Z., Wu C., Zhao H., Wang J., Wang H., Yan Y., Zhong N. (2023). Piezo-photocatalysts in the Field of Energy and Environment: Designs, Applications, and Prospects. Nano Energy.

[42] Bai Y., Zhao J., Lv Z., Lu K. (2020). Enhanced Piezocatalytic Performance of ZnO Nanosheet Microspheres by Enriching the Surface Oxygen Vacancies. J. Mater. Sci..

[43] Alghazeer R., Elmansori A., Sidati M., Gammoudi F., Azwai S., Naas H., Garbaj A., Eldaghayes I. (2017). In vitro Antibacterial Activity of Flavonoid Extracts of Two Selected Libyan Algae Against Multi-drug Resistant Bacteria Isolated From Food Products. J. Biosci. Med..

[44] Kartik T., Piu D., Moni B.S. (2022). Hydrogen Peroxide–assisted Photocatalytic Dye Degradation Over Reduced Graphene Oxide Integrated ZnCr_2_O_4_ Nanoparticles. Environ. Sci. Pollut. Res. Int..

[45] Ma B.C., Ghasimi S., Landfester K., Vilela F., Zhang K.A.I. (2015). Conjugated Microporous Polymer Nanoparticles with Enhanced Dispersibility and Water Compatibility for Photocatalytic Applications. J. Mater. Chem. A.

